# Alpha-Gal Syndrome in Children: Peculiarities of a “Tick-Borne” Allergic Disease

**DOI:** 10.3389/fped.2021.801753

**Published:** 2021-12-23

**Authors:** Francesca Saretta, Mattia Giovannini, Francesca Mori, Stefania Arasi, Lucia Liotti, Luca Pecoraro, Simona Barni, Riccardo Castagnoli, Carla Mastrorilli, Lucia Caminiti, Gian Luigi Marseglia, Elio Novembre

**Affiliations:** ^1^Pediatric Department, Latisana-Palmanova Hospital, Azienda Sanitaria Universitaria Friuli Centrale, Udine, Italy; ^2^Allergy Unit, Department of Pediatrics, Meyer Children's University Hospital, Florence, Italy; ^3^Translational Research in Pediatric Specialties Area, Division of Allergy, Bambino Gesù Children's Hospital (IRCCS), Rome, Italy; ^4^Department of Pediatrics, Salesi Children's Hospital, AOU Ospedali Riuniti Ancona, Ancona, Italy; ^5^Department of Medicine, University of Verona, Verona, Italy; ^6^Maternal and Child Department, ASST Mantua, Mantova, Italy; ^7^Department of Pediatrics, Pediatric Clinic, Fondazione IRCCS Policlinico San Matteo, University of Pavia, Pavia, Italy; ^8^Pediatric Unit and Emergency, University Hospital Consortium Corporation Polyclinic of Bari, Pediatric Hospital Giovanni XXIII, Bari, Italy; ^9^Department of Human Pathology in Adult and Development Age “Gaetano Barresi,” Allergy Unit, Department of Pediatrics, AOU Policlinico Gaetano Martino, Messina, Italy

**Keywords:** food allergy, alpha-gal (α-gal), delayed anaphylaxis, red meat allergy, children, cetuximab

## Abstract

The alpha-gal syndrome is an allergic syndrome that comprises two clinical pictures: an immediate hypersensitivity to drugs containing alpha-gal and a delayed hypersensitivity to the ingestion of red mammalian meat. This allergic syndrome is often under-recognized, and patients are mislabeled with diagnosis as spontaneous urticaria or idiopathic anaphylaxis. Even though less frequently, children could also be of interest, especially in tick-endemic areas. In most cases, a positive anamnesis for tick bites months before the onset of symptoms is recorded. The clinical manifestations could range from asymptomatic cases to severe anaphylaxis. The most frequently used diagnostic test is the determination of specific IgE for alpha-gal. Oral provocation test is usually reserved to unclear cases or to verify tolerance after diet. No long-term follow-up studies have been published, although an elimination diet could lead to a decrease of specific IgE for alpha-gal and a possible reintroduction of some avoided foods. This paper provides a literature review, focused on pediatric age, and an evaluation of available diagnostic tests. We analyze the correlation between tick bites and symptom onset and unfold the different clinical pictures to help clinicians to promptly recognized this syndrome. Lastly, we address unmet needs in this specific allergy.

## Introduction

The alpha-gal syndrome (AGS) is an allergic syndrome comprising two scenarios: an immediate hypersensitivity to drugs containing alpha-gal and a delayed hypersensitivity due to the ingestion of red meat (intended as meat from non-primate mammals). The allergic response observed is due to the production of specific IgE (sIgE) for the disaccharide galactose-α-1,3-galactose (alpha-gal or α-gal), which is expressed on the surface of glycolipids and glycoprotein of mammalian cells and synthesized by the galactosyl-α-1,3-galactosyl-synthetase. In primates, the alpha-gal is not expressed due to a genetic loss-of-function mutation of the GGTA1 gene and is recognized as non-self. Since the 80's, IgG-IgA-IgM for alpha-gal have been described, as a protection against parasitosis and the main cause of xeno-transplant reject reactions (1). It is still not clear what drives the IgE production for alpha-gal (sIgE-α-gal), but in most cases, there is a positive anamnesis for tick bites in the months before the onset of symptoms. It has also been observed that there is a higher incidence of cases in distribution areas of some species of ticks: in the United States of America, “lone star” *Amblyomma americanum*; in Australia, *Ixodes holocyclus*; in Europe, *Ixodes ricinus*; and in Asia, *Ixodes nipponensis*.

## Biological Sources of Alpha-Gal

Alpha-gal is expressed in all non-primate mammals (not only in beef, pork, elk, reindeer, and lamb but also in small mammals such as squirrels or beaver), and it could be found in meats, internal organs, meat by-products, gelatin, milk, and dairy products. Alpha-gal is resistant to heat and pepsinic lysis (2) and is therefore present also in all industrial by-products such as food and drugs and even in snake poison antidotes (3).

Alpha-gal is also found in carrageenan, obtained from boiled red algae (*Chondrus crispus* and *Gigartina mamitiosa*), frequently used in food and drug preparations and identified as E407 preservatives. Carrageenan could be found, e.g., as a clarifying agent, as a hydrating agent for foods, in powdered infant's milk, and in toothpaste.

It has been demonstrated that alpha-gal could also be present in porcine and bovine substitute's heart valves; an allergic sensitization to alpha-gal has been associated to an early valvular deterioration (4) and to peri-operative anaphylaxis after valvular substitution (5). In particular, in subjects younger than 65 years old and sensitized to alpha-gal, an increase of atherosclerosis and plaque instability have been observed (6).

In a recent study (7), an increase of sIgE-α-gal after multiple Hymenoptera stings has been observed with further decrease after 6–8 weeks. This could be due to the increase of *Api m5*, a dipeptidyl-peptidase similar to an enzyme found in tick saliva. Researchers are still investigating if other parasites, such as *Trombicula*, could be a carrier of alpha-gal (8).

## From the First Case Reports to Sensitization and Clinical Manifestations

A detailed chronology of scientific discoveries about AGS starts in 1989, when Mrs. Sandra Latimer and Dr. Antony Deutsch (9) reported in Georgia (USA) the first case reports of delayed red meat allergy with a possible correlation to tick bites. In 2007, Van Nunen et al. (10) reported an Australian case series of delayed red meat allergy, with patients recalling *I. holocyclus* tick bites before symptom onset and having positive prick test or specific IgE to red meat. In the same year, O'Neil et al. (11) observed a higher frequency of cetuximab hypersensitivity reactions (HRs) in south-east of USA, arguing firstly a possible role of specific IgE toward cetuximab, and Qian et al. (12) demonstrated alpha-gal in F portion of cetuximab. In 2008, Chung et al. (13) found positive sIgE cetuximab in cetuximab-allergic patients before drug infusion, and in 2009, Commins et al. (14, 15) published a case series of delayed red meat allergy in areas with overlapping cetuximab HR reports and demonstrated the correlation of these reactions to previous tick bites and further increase of sIgE-α-gal (interestingly at first in researchers and then in patients). *A. americanum* was found responsible for those sensitization. Finally, in 2013, Hamsten et al. (16) demonstrated the presence of alpha-gal in tick saliva and the correlation between tick bites and following sensitization to alpha-gal.

Some mechanisms have been proposed to explain how alpha-gal enters in tick intestine: the tick itself produces the alpha-gal, the alpha-gal comes from a previous tick meal on a carrier animal, and the alpha-gal is expressed by some symbionts in tick saliva (17). Another unclear question is how the tick bite induces sIgE-α-gal production. Some hypotheses have been suggested:

1. The alpha-gal in tick saliva, once inoculated, interacts with antigen-presenting cells (APC) and B cells, directly stimulating sIgE-α-gal production.
2. Tick bite inoculates the alpha-gal and some adjuvants, which drive the isotypic B cell switch from IgG/IgM-α-gal to sIgE-α-gal (18).
3. Tick bite induces a skin barrier trauma or interferes with skin microbioma, causing the production of pro-allergic molecules, which drive the isotypic B cell switch (19).

However, skin trauma plays a pivotal role in altering the micro-environment, favoring the switch toward IgE and Th2 immunity by promoting the release of TSLP, IL25, and IL33 (20, 21). Basophils could also play a role, as some studies have demonstrated (22–24).

## The Delayed Timing in Alpha-Gal Food Allergy

**Figure 1:**
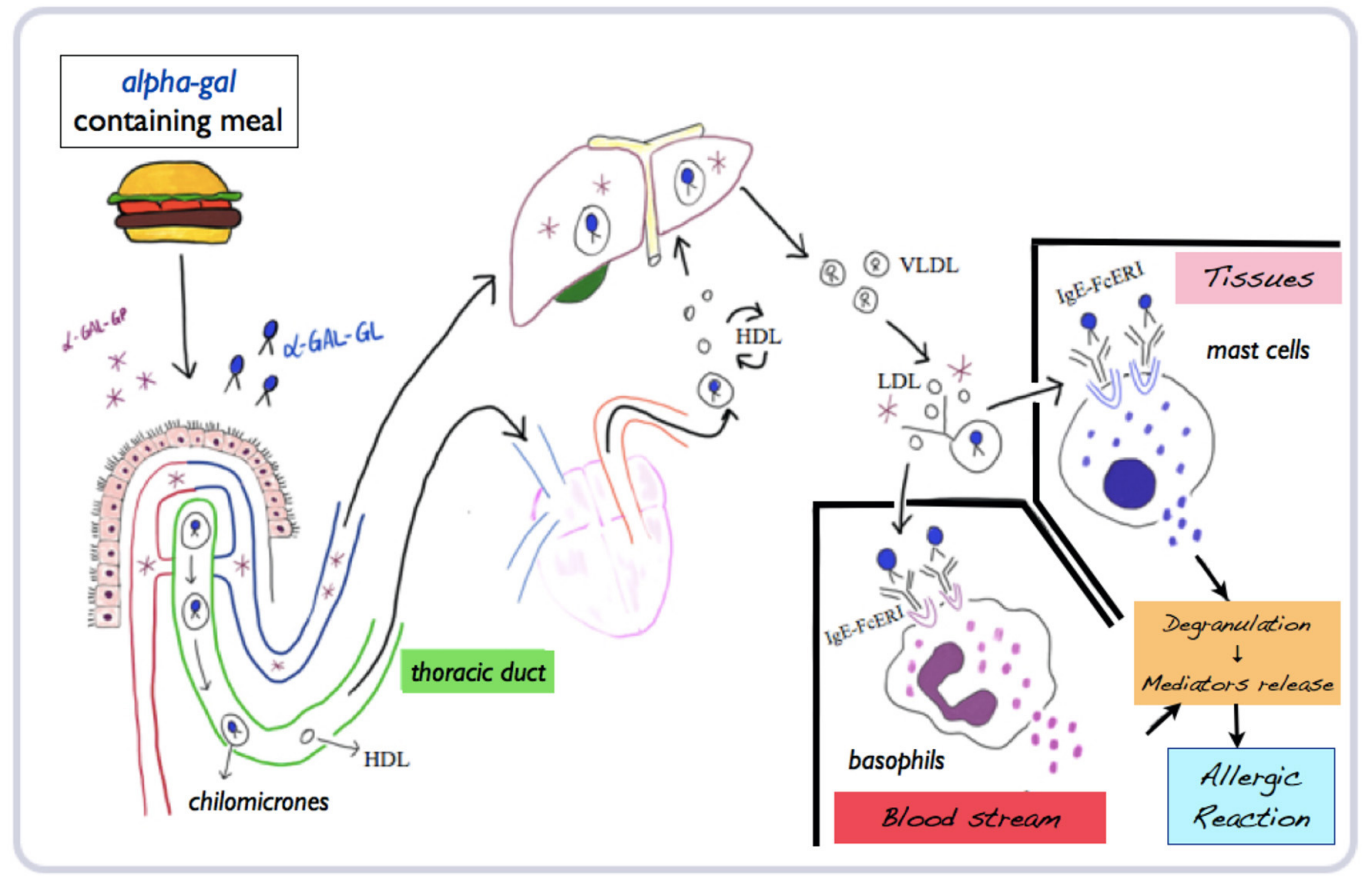
Mechanisms of absorption, processing, and metabolization of alpha-gal and subsequent allergic reaction. Adapted and modified from Wilson and Platts-Mills (26) (mod.) (α-gal-GP, glycoproteins; α-gal-GL, glycolipids; HDL, high-density lipoproteins; LDL, low-density lipoproteins; VLDL, very-low density lipoproteins).

Once ingested through a meal with red meat products or by-products, glycolipids and glycoproteins expressing alpha-gal are absorbed in the gastrointestinal tract, through chylomicrons entering the lymphatic system (thoracic duct) and systemic circulation (superior vena cava). In the following 2–4 h, chylomicrons reduce their diameter from 400 to 20 nM in low-density lipoproteins. With this diameter, they could cross skin interstitial tissues or vessel walls and activate mast cells and basophils. Once cross-linked with IgE–FcεRI complex on basophils and mast cells, which express the same high-affinity IgE receptor, degranulation starts with subsequent IgE-mediated reaction. The whole interval time needed for these processes could therefore explain the delay of symptoms in AGS by food ingestion (25, 26) (Figure 1).

## Clinical Manifestations of AGS

Sensitization usually occurs through inoculation of alpha-gal *via* tick bites, which could lead to the following:

### Asymptomatic Sensitization

A low-grade sensitization to alpha-gal is not always correlated with clinical manifestations. In a study on German adults (27) with occupational risk for tick bites (hunters and rangers), 35% of patients had sIgE-α-gal > 0.10 kUI/l and 19.3% had sIgE-α-gal > 0.35 kUI/l. Only five patients, though, referred allergic symptoms after red meat ingestion, all with sIgE-α-gal > 0.35 kUI/l (8.6% total). Importantly, no differences, in terms of demographics and clinical data, were found, within the sIgE-α-gal > 0.35 kUI/l patient group, between patients who reported meat allergy or not. Interestingly, four out of five of these patients were diagnosed with AGS while participating in the study (27).

### An Allergic Reaction After Ingestion of Food Containing Alpha-Gal

This allergy has unusual characteristics: (a) symptoms are delayed (often after 3–6 h as a timing of onset) but clinically immediate; (b) it does not appear after every ingestion; and (c) it could start years after sensitization and for food previously tolerated (28). A wide intra-individual variability of the trigger dose has been observed (probably due to allergic co-factors and different alpha-gal quantities in food). Patients with mastocytosis seem to react to lower doses (17).

### Allergic Reaction to Drugs and Vaccines Containing Alpha-Gal

Parenteral administration is the main sensitization form, with immediate reactions. In AGS, the most frequent culprit drug is cetuximab, a monoclonal antibody prescribed for colorectal cancer. Vaccines containing gelatin could be responsible for allergic reactions, since gelatin present in vaccines is usually pork derived (29). Therefore, patients must be instructed to check vaccine composition in order to avoid allergic reactions.

### Allergic Reactions After Further Tick Bites

Repeated tick bites could lead to local and systemic reactions such as anaphylaxis. Tick bite anaphylaxis, typically severe, has been reported mainly in an endemic area in Australia, and it is more frequent in adults and if the tick is removed or tingled, and it is preventable if the tick is killed *in situ* (30).

## Clinical and Allergological Characteristics of AGS Pediatric Patients

### Mixed Adult/Children Studies

The study by Mabelane et al. (31) included 131 subjects from a South African rural area (age 4–65 years old; 65% > 13 years old), all referring to delayed red meat reactions. In 114 patients, an oral provocation test has been performed (positive in 71.1%), and no reactions were recorded in control subjects with positive sIgE-α-gal. In the patient group, two different phenotypes have been observed: 21%, mostly women, complained only abdominal cramps; 79% showed different combinations of abdominal symptoms (77.7%), skin and mucous membranes reactions (53.1%), and respiratory or hypo-perfusion symptoms (4.9%). No significant differences were observed in regard to age, symptom onset, alpha-gal sensitization, and sIgE-α-gal/total sIgE ratio.

Wilson et al. (32) have recently confirmed these data in another mixed adult/children population. In 35 children (5–18 years old) with delayed red meat reactions (89%, >2 h after ingestion), 97% showed sIgE-α-gal > 0.35 kUI/l, and 100% recalled a tick bite in the previous 10 years. Among the children, 89% had urticaria, 49% had anaphylaxis, 66% had gastrointestinal symptoms, and 51% required ER evaluation. In this population, higher levels of sIgE-α-gal were observed in urticaria/anaphylaxis compared to isolated gastrointestinal symptoms (*p* = 0.002). It may therefore be possible that isolated abdominal pain is an under-reported and under-diagnosed feature of AGS, as also reported by other authors (33).

### Pediatric Studies

In 2013, the first pediatric study has been published by Kennedy et al. (34). Fifty-one children (4–17 years old) with delayed red meat allergic reactions (92% urticaria, 31% angioedema, 64% gastrointestinal symptoms, and 44% anaphylaxis) have been analyzed. Most of them (88%) showed positive sIgE-α-gal, half required ER evaluation, and 19% required adrenaline treatment. All parents recalled a tick bite before symptom onset, and 87% remembered the tick bite as persistently itching and hyperemic.

Donaldson and Le (35) reported a 3.3% prevalence of AGS in a 5-year period (2014–2018) in children from Missouri with food allergy diagnosis. Children presented urticaria in 78%, anaphylaxis in 29%, angioedema in 21%, and gastrointestinal symptoms in 17% of cases, and ER evaluation was required in 32% of cases.

Clinical and allergological characteristics of the pediatric case series are briefly reported in Table 1. Other case reports of AGS in childhood have been published (36–40).

**Table 1:** Clinical and allergological characteristics of an AGS pediatric case series.

**Author**	***n***	**Age**	**Total IgE**	**sIgE α-gal**	**Clinical manifestations (%)**				**ER%**	**Ref**
		**Years**	**kUI/l**	**kUI/l**	**Urticaria**	**Angioedema**	**GI**	**Anaphylaxis**		
Wilson et al.	35	13	170	8.2	93	–	66	49	51	(32)
Kennedy et al.	51	12	147	8	92	31	64	44	50	(34)
Donaldson and Le	42	9.95	–	2.9	78	21.9	17	29.2	32	(35)

*Ref, references, GI, gastrointestinal, ER, emergency room access*.

### Adult/Children Differences

AGS shows different characteristics in children compared to adults. Children seem to have a lower incidence of AGS [13% in the study by Wilson et al. (32) and 35% in the study by Mabelane et al. (31)], lower sIgE-α-gal levels (although there is no difference in reaction timing or nature or exposure to tick bites), a restricted number of trigger foods, a predominance of gastrointestinal symptoms, and exercise as the main allergic co-factor (7).

### Risk Factors

Some risk factors have been associated with a higher risk of developing AGS (7, 41):

- Outdoor works and hobbies (forest guards, hunters, and hikers) and environmental conditions that increase the development and spread of ticks;
- Non-B blood types;
- Allergic co-factors (e.g., drugs, alcohol, and exercise);
- Age: possibly correlated to the studied population, though, e.g., in the study of Mabelane et al. (31) patients have a younger age probably due to an earlier environmental exposure;
- Atopy: the association has not been clarified yet (42);
- Cat allergy: alpha-gal epitope is also present on cat, implying a possible association between AGS and cat dander sensitization. However, not all studies agree on this hypothesis (43);
- Meat type (giblets vs. lean cut): Morisset et al. (44) demonstrated a correlation between meat type and inhibitory properties of alpha-gal, showing that the kidney has a higher alpha-gal content;
- Type of exposure: parenteral exposure is correlated with more severe reactions compared to ingestion.

### Protective Factors

B/AB blood types seem to act as a protective factor (41). This is explained looking at the similarity between alpha-gal and B antigen in ABO system: the only difference is a fucose residue on B antigen. Many studies have underlined that B or AB blood type subjects are under-represented in AGS case series, with lower levels of sIgE-α-gal production (45–47).

## Diagnosis

As in adults, the diagnosis is based on clinical history and allergy tests.

### Clinical History

1. Clear up when signs and symptoms appear: delayed reactions begin at least 2 h after meat ingestion, while parenteral administration evokes immediate reactions;
2. Ask for previous tick bites and bite reactions (as persistent itchiness or redness);
3. Evaluate the number and characteristics of episodes;
4. Check for possible allergic co-factors;
5. Think about AGS in any allergic reaction without apparent cause. In a study on teenagers and adults (48), initially classified as idiopathic anaphylaxis, AGS was the definitive cause in 33%;
6. As suggested by Kennedy et al. (34), in children, especially those older than 5 years old, with delayed reaction to red meat or dairy products previously tolerated, AGS must be excluded.

### Allergy Tests

No pediatric studies have specifically evaluated sensitivity and specificity of allergy tests, and most of them rely on the same positivity cut-off for skin tests and sIgE-α-gal.

Commercial prick tests for red meat showed a low sensibility and are not recommended (14). Cooked red meat, gelatin, and cetuximab prick-by-prick seem to be more useful (14, 49). In case of isolated beef meat prick positivity, milk proteins must be checked to exclude a milk allergy (50). In a study by Kennedy et al. (34), milk protein positivity in children with no history of milk allergy and who currently eat milk and dairy products without reactions has been reported. Intradermal tests with red meat extracts are not recommended for an elevated anaphylaxis risk. Some studies suggest more reliable results with gelatin intradermal test (49, 51–53).

Most allergological clinics rely on specific IgE for alpha-gal. Commercially available kits are Phadia-Thermo Fisher in Europe and Viracor-Eurofins in the United States. It is recommended to evaluate both sIgE-α-gal and total IgE and calculate the ratio. Scientific experts suggest to consider as positive an sIgE-α-gal level > 0.1 kUI/l (92.3% specificity, 100% sensibility in patients with clinical AGS) (7). However, the most accurate diagnosis is performed with sIgE-α-gal > 2 kUI/l and sIgE-α-gal/IgE total ratio > 2% (54). Correlations have been demonstrated neither between sIgE-α-gal and severity of reactions (7) nor between sIgE-α-gal and reaction timing (32). Commins (7) recommends to test sIgE for milk proteins, cat dander, red meat (beef or pork), and beef/pork gelatin, especially in patients with positive sIgE-α-gal and negative clinical history or in patients clinically suspected with negative sIgE-α-gal (54). In this case, sIgE-α-gal for cat serum albumin (Fel d 2) must be checked to identify cat-pork syndrome (7).

Recently, the basophil activation test has shown a possible usefulness in distinguishing symptomatic patients with AGS from those with laboratory-isolated sensitization (55).

Oral provocation test could be useful to confirm AGS diagnosis in doubtful cases or asymptomatic sensitization, but it is not standardized yet, especially in regard to the quantity and quality of meat to be tested. Morisset et al. (44) designed a 3-day protocol with increasing doses of pork kidney, reaching a cumulative dose of 965 mg at day 1, 7 g at day 2, and 150 g at day 3. Mabelane et al. (31) performed an oral provocation test with beef sausages (one sausage of 63 g for body weight <30 kg, two sausages for weight 30–60 kg, and three sausages for weight >60 kg).

## Management

Once the AGS diagnosis is clear, it is important to discuss with the patient about diet adjustment, further tick bite avoidance, and treatment plan.

Elimination diet could be set upon clinical history and comprises the elimination of all mammalian meats. The patient should be taught on label reading and instructed to avoid meat and by-products, especially internal organs (spleen and liver), which could be used as an ingredient or as delicatessen, and fat by-products (lards, ready-to-use broth, and gelatin). In some cases, it could also be necessary to avoid dairy products (56).

Additional tick bites must be avoided since they could further increase sIgE-α-gal and the risk of allergic reactions to previously tolerated foods. The patient and family should be instructed on clothing type, repellent products, and the recognition and prompt removal of ticks.

Regarding treatment, the symptoms, even if delayed, must be treated as in immediate reactions. For mild reactions, oral antihistamine and steroids could be used. For severe reactions and anaphylaxis, auto-injectable adrenaline must be prescribed. An oral desensitization case report (37) on a 10-year-old boy, with two previous anaphylaxis, has also been published. Oral provocation test was performed, with a reaction after ingestion of 4 g of cooked beef. A 24-day desensitization protocol has allowed to reach 120 g of cooked beef, to be ingested daily to maintain acquired tolerance.

## Prognosis

Nowadays, there are no prospective studies on AGS natural history. sIgE-α-gal levels often decline spontaneously in those who avoid tick bites (14). This decline has been demonstrated by Kim et al. (57), who also noticed that sIgE-α-gal increases after repeated tick bites but not always with an isolated single bite. The authors underlined that it is important to periodically evaluate sIgE-α-gal. After 12 months, if a reduction of sIgE-α-gal < 0.35 kU/l or < 2% of total IgE is detected, in the absence of accidental ingestion, a gradual reintroduction of low-fat meat could be proposed.

## Unmeet Needs

The rarity of AGS, the diagnostic delay, and the possible under-diagnosis of this allergy suggest that more studies are needed. In particular, the following are suggested:

- Epidemiological population study (ASG prevalence in subjects with meat allergy, other food allergies, and a concurrent blood type characterization)
- Determination of allergen content in different foods and drugs
- Analysis of the molecular mechanisms of delayed timing
- Evaluation of allergic co-factors and dose–response effect
- Evaluation of the cooking process and the role of other ingredients and environmental factors
- Long-term follow-up (feasibility and usefulness of early detection of sensitization)
- Evaluation of high-risk patient screening
- Standardization of oral provocation test and desensitization protocols
- Standardization and data collection on sensitivity and specificity of diagnostic tests in pediatric age.

## Conclusions

AGS is a rare, atypical, and under-rated allergy, less frequent in childhood but involving those living in tick-endemic areas. Red meat ingestion usually not only causes delayed reactions (2–6 h after meal) but could also lead to severe reactions. Diagnosis relies on clinical history and sIgE-α-gal determination. It is important to evaluate for AGS every patient with a history of idiopathic urticaria or anaphylaxis and unclear allergic reactions with unusual timing or characteristics. More have to be discovered on natural history, but long-term management is based on solely elimination diet and tick bite avoidance.

## Author Contributions

EN conceived the study and supervised it. FS wrote the manuscript. All the authors performed the research, selection of the sources, contributed to the article, and approved the submitted version.

## Funding

The publication fee was financed by the Italian Society of Pediatric Allergy and Immunology. However, no significant funding source could have influenced the outcomes of this work.

## Conflict of Interest

The authors declare that the research was conducted in the absence of any commercial or financial relationships that could be construed as a potential conflict of interest.

## Publisher's Note

All claims expressed in this article are solely those of the authors and do not necessarily represent those of their affiliated organizations, or those of the publisher, the editors and the reviewers. Any product that may be evaluated in this article, or claim that may be made by its manufacturer, is not guaranteed or endorsed by the publisher.
